# Key Anthropometric and Physical Determinants for Different Playing Positions During National Basketball Association Draft Combine Test

**DOI:** 10.3389/fpsyg.2019.02359

**Published:** 2019-10-22

**Authors:** Yixiong Cui, Fuzheng Liu, Dapeng Bao, Haoyang Liu, Shaoliang Zhang, Miguel-Ángel Gómez

**Affiliations:** ^1^AI Sports Engineering Lab, School of Sports Engineering, Beijing Sport University, Beijing, China; ^2^China Institute of Sport and Health Science, Beijing Sport University, Beijing, China; ^3^School of Medical Humanities, Capital Medical University, Beijing, China; ^4^Facultad de Ciencias de la Actividad Física y del Deporte–INEF, Universidad Politécnica de Madrid, Madrid, Spain

**Keywords:** agility, speed, leg-power, NBA, talent identification

## Abstract

Annual draft combine test of National Basketball Association (NBA) is a key player testing process where prospective players with extraordinary athletic abilities are evaluated and the assessment results would further inform the determination of prospective players for the league during draft day. Nonetheless, key attributes from the combine test that distinguished successful players in the draft from those unselected has yet to be investigated. The study was aimed to: (i) compare the difference between NBA drafted and undrafted players from five playing positions, considering anthropometric characteristics and physical fitness ability during draft combine test; and (ii) determine the key combine test factors that most effectively discriminate between draft groups. A total of 3,610 players participating in the 2000–2018 NBA draft combine test were included. Independent *t-*test was applied to compare difference between drafted and non-drafted players in variables related to anthropometrics, and strength and agility test. A descriptive discriminant analysis was subsequently used to identify which variables could best discriminate between two draft groups in each playing position. The significance level was set at *p* < 0.05. The drafted players from five positions outperformed the undrafted in height, wingspan, vertical jump height and reach, line agility and three-quarter sprint test (*p* < 0.01, ES = 0.26–0.87). The discriminant functions for each position (*p* < 0.001, Λ = 0.81–0.83) were emphasized by specific variables that discriminated both draft groups. The findings revealed that in addition to height and wingspan, leg power served as key determinants for being drafted as guards, as did agility and speed for power forwards and centers.

## Introduction

Generally, the athletic ability of basketball players is a multi-dimensional competence constituted by a series of factors, such as morphological characteristics, physical fitness, technical and tactical skills, or mental abilities (Ostojic et al., 2006; Köklü et al., 2011; Ferioli et al., 2018). This ability is positively associated with players’ future career achievements and success within highly competitive level (Hoffman et al., 1996; Berri et al., 2011; Teramoto et al., 2018). Among all factors related to athletic ability, morphological features is placed at the primary place during players’ evaluation and selection (Vaquera et al., 2015). Particularly, height and weight would be critical when establishing players’ on-court positions (Dežman et al., 2001). Together with upper limb length and standing reaching height, these factors could influence players’ match performance in game situation and predict whether these athletes will reach the top level in basketball (Apostolidis and Emmanouil, 2015; Garcia-Gil et al., 2018).

Whilst due to the physically demanding characteristic of basketball, players are required to execute various high-intensity actions and quick reactions within short interval of periods during the game, like sprinting, dribbling, shuffling, jumping and fast change of directions (Ben Abdelkrim et al., 2010; Alemdaroğlu, 2012). Therefore, the physical fitness is usually considered as an essential factor that determines the development of other athletic abilities to a large extent. Because all those functional, technical and tactical activities are made possible due to elite players’ muscular power of upper and lower limb, speed, agility and coordination at elite level (Hoare, 2000; Ostojic et al., 2006; Fort-Vanmeerhaeghe et al., 2016). Indeed, these fitness abilities are considered more important for high-level basketball players due to the fact that aerobic fitness level is not a strong discriminant characteristic between professional and semi-professional players (Köklü et al., 2011; Ferioli et al., 2018), and success in basketball is more dependent on players’ anaerobic power (Hoffman and Maresh, 2000).

Previous studies have designed various physical fitness tests to assess these specific muscular performance of basketball players (Hoffman et al., 1996; Erculj et al., 2010; Alemdaroğlu, 2012; Conte et al., 2018). For instance, via vertical jump test, it is found that vertical jump heights were similar between different playing positions, and elite basketball player would have standing vertical jump values higher than 60 cm and maximal vertical values higher than 80 cm (Ostojic et al., 2006; Mehran et al., 2016). Moreover, professional forwards and guards showed superior sprinting and agility performance than centers (Köklü et al., 2011).

The National Basketball Association (NBA) brings together the best men’s basketball players from all over the world and is also considered as the most competitive men’s basketball league worldwide (Sampaio et al., 2015). Since 2000 in Chicago, it annually hosts a draft combine that includes a series of standardized measurements and performance testing programs in order to help executives of the NBA teams to evaluate and identify prospect talents. Within the combine test, anthropometric, athletic abilities and shooting skills of players will be examined and information gained during this process will be critical for decision-makers to later pick those meeting their needs. However, research on the NBA draft has been relatively scarce. Mehran et al. (2016) found similar combine test performance between players who previously suffered from anterior cruciate ligament injuries and those without knee injury. The study by Teramoto et al. (2018) implied that length-size and upper-body strength demonstrated during draft combine were more highly related to the future on-court performance. Little is known concerning the difference in test results between drafted players and those unselected. Besides, more evidence is needed to confirm which combine test factors could effectively predict successful draft if game-specific positions were accounted for. This may allow for a better understanding about the draft data and set realistic benchmarks for other leagues that strive to reach NBA level.

Based on this rationale, the purpose of this study was twofolds, (i) to investigate the difference between NBA drafted and undrafted players from five playing positions (point guard, shooting guard, small forward, power forward and center), considering anthropometric characteristics and physical fitness ability during draft combine test; and (ii) to determine the key combine test factors that most effectively distinguish both draft groups. According to the existing literature, it was hypothesized that drafted players would outperform undrafted in all test-related statistics, except for body fat and there would be position-specific predictors to draft result.

## Materials and Methods

### Subjects

In the study, basketball players who participated in the NBA Draft Combine from 2000 to 2018 were sampled as subjects. All participating players were required to have at least an age of 19 years during the draft year (Teramoto et al., 2018). Normally, the Combine contains the following tests: anthropometric measurement, strength and agility test, non-stationary and spot up shooting tests. However, due to the fact that detailed data of shooting tests from 2000 to 2013 are not available^1^ (accessed in 22/12/2018), the study only considered the former two tests. Data of test results were made available by Beitai Digital China Company (Beijing). The Company maintained the anonymity of player complying with European Data Protection Regulation. In total, 3610 player observations were included (drafted: 1,160, undrafted: 2,450). The study was approved by the research commission of Beijing Sport University with Approval No.: 2019068H and all procedures are conducted following the European General Data Protection Law in order to maintain the anonymity of sampled players.

### Procedures and Variables

All players were divided into five playing positions according to their position information registered during the draft^2^ (accessed in 11/12/2018): point guard (PG, *n* = 691), shooting guard (SG, *n* = 861), small forward (SF, *n* = 851), power forward (PF, *n* = 851) and center (C, *n* = 560). If a player was identified with two playing position (e.g., PG-SG or SG-SF), we just classified him based on the first position. The average ages (years) for drafted (*n* = 184) and undrafted (*n* = 507) PG are 20.4 ± 1.22 vs. 22.1 ± 0.97; for drafted (*n* = 270) and undrafted (*n* = 591) SG: 20.7 ± 1.37 vs. 22.1 ± 0.93; for drafted (*n* = 253) and undrafted (*n* = 394) SF: 20.8 ± 1.49 vs. 22. 3 ± 1.18; for drafted (*n* = 270) and undrafted (*n* = 581) PF: 20.7 ± 1.54 vs. 22.2 ± 1.16; for drafted (*n* = 183) and undrafted (*n* = 377) C: 20.5 ± 1.54 vs. 22.1 ± 1.3.

The drafted players were significantly younger than the undrafted counterparts (*p* < 0.01, *z* = −8.305 to −6.93, *r* = −0.63 to −0.43, medium to large effect sizes).

As previously noted that the current study analyzed the anthropometric characteristic and strength and agility test performance within NBA Draft Combine, fourteen combine measurement variables from these two categories were selected. The anthropometric variables were: height without shoes, weight, wingspan, standing reach, body fat percentage; while the strength and agility variables were: no step vertical jump, no step vertical reach, max vertical jump, max vertical reach, bench press, lane agility and three-quarter court sprint. Before starting the strength and agility testing, players always warm up and stretch for 10–15 min and receive proper instruction and a demonstration of each test. When the testing begins, it follows the order of no-step vertical jump, maximum vertical jump, lane agility, three-quarter sprint and bench press, and the rest intervals between tests are at least 2 min. The detailed explanation of these variables is shown in Table 1. Additionally, those variables originally in United States customary units (feet/inches/pounds) were converted into equivalent International System of units (cm/kg). The existing literature showed that these tests and measurements were selected by NBA conditioning coaches and were valid and reliable methods to evaluate anthropometric and physical characteristic that are vital for basketball-specific performance (Foran and Pound, 2007; Ferioli et al., 2018; Teramoto et al., 2018).

**TABLE 1:** List of NBA combine measurement variables, definition, and measurement protocol.

**Combine measurement variable**	**Definition and measurement protocol**
Height without shoes	Height is measured in feet and inches using a physician scale, while the player is not wearing shoes.
Weight (kg)	Body weight is measured using a physician scale
Wingspan	The tip of the left hand to the tip of the right hand is measured in feet and inches using a measuring tape, while the player is stretching the arms horizontally.
Standing Reach	The player is standing straightly and reaching both arms up, making them vertical to the floor, while their longest tip of their hands to the floor is measured using a measuring tape.
Body fat Percentage	Body fat percentage is assessed by measuring the skinfold thickness of pectoral, abdomen, and quadriceps using a skinfold caliper.
Hand Length (cm)	The length from the bottom of player’s palm to the tip of the middle finger measured with a measuring tape.
Hand Width (cm)	The length from the tip of player’s thumb to the tip of the small finger measured with a measuring tape.
No Step standing vertical Jump (cm)	Without running, the player starts with both feet flat on the floor and jumps vertically as high as possible and taps the Vertec device. And then the difference between the standing reach and the no step vertical reach measured is measured.
No Step Vertical Reach (cm)	The height of Vertec device a player reaches when executing no step jump.
Maximum Vertical Jump (cm)	With running, the player jumps vertically as high as possible and taps the Vertec device. The player can take any number of steps as long as the approach distance is between the free-throw line and a 15-foot (4.6 m) arc, and can choose either a one-foot or two-foot takeoff. The difference between the standing reach and the maximum jump reach is measured.
Maximum jump reach (cm)	The height of Vertec device a player reaches when executing maximum vertical jump.
Bench Press	The total number of completed repetitions a player has while performing 83.9 kg bench press with a standard, proper technique.
Lane Agility	A cone is placed at each of four corners of the lane. Starting from the left corner of the free-throw line, the player runs forward to the baseline, side-shuffle to the right corner of the lane, backpedal to the right corner of the free-throw line, and side-shuffle to the left to go back to the starting point. Then, the player changes the direction, side-shuffle to the right corner of the free-throw line, runs forward to the baseline, side-shuffle to the left corner of the lane, and backpedal to go back to the starting point. The score is the time to cover the distance measured in seconds.
Three-quarter court sprint (*s*)	Two cones are placed at corners of the lane along the baseline, and other two cones are placed at the corners of the opposite free-throw line. The player sprints from the baseline to the three-quarter length of the court as fast as possible, and their time is measured.

### Statistical Analyses

Data of analyzed combine measurement variables are presented as mean and standard deviation (SD). After testing data normality distribution (Shapiro–Wilks test) and equal variances of compared variables, the independent *t-*test was applied to investigate difference between drafted and undrafted players in these variables, regarding five playing positions (PG, SG, SF, PF, and C). While Man–Whiteney *U* test was run for other variables when the assumption of homogeneity of variances is violated. In order to interpret the meaningfulness of differences, the Cohen’s *d* was used as effect size statistics for *t*-test and was calculated and interpreted according to the following thresholds: 0.2, trivial; 0.6, small; 1.2, moderate; 2.0, large; 4.0, very large; and ≥4.0, extreme large (Hopkins et al., 2009). Meanwhile *r* was used as effect size for Man-Whiteney *U* test and interpreted for *t*-test according to the following thresholds: 0.3, small; 0.50, moderate; ≥0.5, large (Cohen, 1988).

**FIGURE 1:**
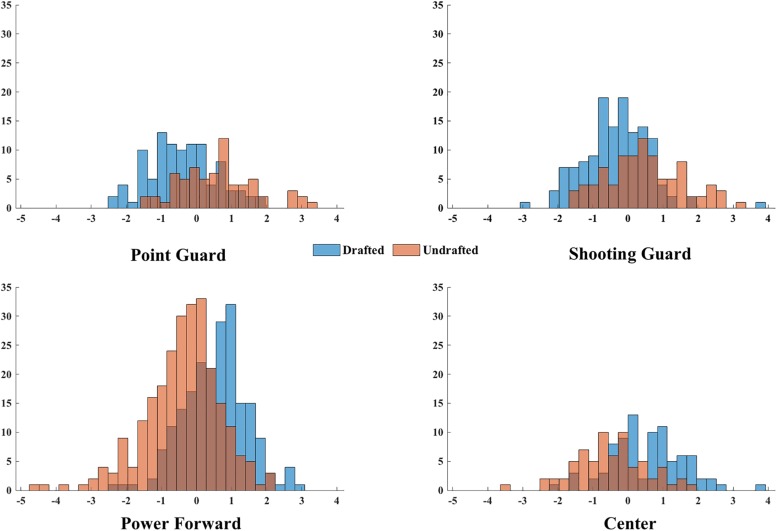
Distribution of discriminant scores across for point guards, shooting guards, power forwards and centers.

Based on the results of previous test, we selected the variables that are shown to be significantly different between drafted and undrafted players within each playing position. Afterward, a descriptive discriminant analysis was used to identify which measurement variables could best discriminate drafted and undrafted players. The effect sizes of the discriminant functions were assessed by squared canonical correlation ($r_{c}^{2}$), which explains the variance associated with each function; and partial eta square ${\text{η}}_{\text{p}}^{2}$, which describes the variance for the entire analysis (Leech et al., 2014). The thresholds for the interpretation of $r_{c}^{2}$ magnitude was as follows: 0.09, small; 0.25, moderate; and ≥0.25, large; while the strength of ${\text{η}}_{\text{p}}^{2}$ was interpreted by the following thresholds: 0.06, small; 0.14, moderate; and ≥0.14, large (Cohen, 1988). Moreover, a variable would be regarded as meaningful contributor to the differentiation of two groups if its structure coefficients (SC) were higher than |0.30|. Validation of discriminant models was applied using the leave-one-out method of cross-validation, which takes subsets of data for training and testing, and is necessary for understanding the usefulness of discriminant functions when classifying new data. The analyses were performed using IBM SPSS software (Armonk, NY, United States: IBM Corp.), and the significance level was set at *p* < 0.05. Finally, Matlab^®^ (MathWorks, Inc., Massachusetts, United States) dedicated routines were used to visualize the discriminant function plots using discriminant scores, which are illustrative of how drafted and undrafted players were separated by the obtained functions (Figure 1).

## Results

The means and standard deviations of combine measurements variables are presented in Table 2 along with the comparison results between drafted and undrafted players from five playing positions. It is shown that there were significant differences between drafted and undrafted players of all positions in all variables except hand length, hand width and bench press. Compared to undrafted players, the drafted from five positions were taller, had longer wingspan, had better performance in no step standing vertical jump test, no step vertical reach test and maximum vertical jump test, and ran faster in lane agility test and three-quarter court sprint test (*p* < 0.01, Cohen’s *d* = 0.26–0.74 and *r* = 0.15–0.39, small to moderate effect size).

**TABLE 2:** Descriptive statistics and comparisons of combine test measurements for drafted and undrafted players from five playing positions within 2000–2018 NBA combine draft.

**Position**	**PG**			**SG**			**SF**			**PF**			**C**		
**Result**	**Drafted**	**Undrafted**	**Effect**	**Drafted**	**Undrafted**	**Effect**	**Drafted**	**Undrafted**	**Effect**	**Drafted**	**Undrafted**	**Effect**	**Drafted**	**Undrafted**	**Effect**
										
**N range:**	**85–183**	**54–501**	**Size**	**120–270**	**82–588**	**Size**	**86–252**	**48–387**	**Size**	**108–269**	**74–576**	**Size**	**70–183**	**45–377**	**Size**
										
**Variables (unit)**	***M* (*SD*)**	***M* (*SD*)**		***M* (*SD*)**	***M* (*SD*)**		***M* (*SD*)**	***M* (*SD*)**		***M* (*SD*)**	***M* (*SD*)**		***M* (*SD*)**	***M* (*SD*)**	
Height (cm)	186.3 (5.4)	183.9 (5.4)	0.45**	192.5 (4.5)	189.9 (4.3)	0.58**	198.1 (5)	196.8 (5)	0.26**	203.5 (3.9)	201.6 (3.6)	0.50**	207.9 (3.9)	206.5 (4.3)	0.34**
Weight (kg)	85.4 (6.8)	81.8 (7.3)	0.50**	91.1 (6.5)	87.7 (8)	0.19**	97 (6.9)	94.4 (8.5)	0.16**	105.9 (8.2)	102.3 (9.5)	0.19**	111.5 (8.9)	110.2 (12.4)	0.06
Wingspan (cm)	198.0 (7.3)	193.4 (6.8)	0.65**	204.5 (6.2)	199.9 (6.3)	0.74**	209.9 (6.6)	207.6 (6.6)	0.34**	216 (5.6)	213.2 (6.1)	0.49**	221.4 (7.3)	217.7 (6.1)	0.55**
Standing Reach (cm)	247.8 (8.2)	244.6 (8)	0.40**	256.4 (6.7)	252.6 (6.6)	0.57**	264.1 (7.6)	263.3 (7.2)	0.10	272 (5.5)	269.4 (5.7)	0.46**	278.2 (6.4)	276.2 (6.3)	0.32**
Body Fat (%)	6.1 (1.8)	7.4 (2.5)	0.27*	6.5 (2)	7.1 (2.6)	0.16**	6.8 (1.8)	7.6 (2.5)	0.19*	8.2 (2.9)	8.8 (3.4)	0.08	8.8 (3.7)	10.2 (4.1)	0.34*
Hand Length (cm)	21.2 (1.0)	21.1 (1.5)	0.08	21.9 (0.9)	21.6 (1.3)	0.28	22.4 (1)	22.5 (1.4)	0.10	22.8 (1.1)	23 (1.4)	0.09	23.6 (1.3)	23.4 (1.5)	0.12
Hand Width (cm)	22.9 (1.4)	22.5 (1.7)	0.28	23.6 (1.6)	23.4 (1.6)	0.08	23.9 (1.6)	24 (1.3)	0.09	24.4 (1.8)	24.3 (1.9)	0.04	25.2 (1.7)	25.2 (2.0)	0.02
Standing Jump (cm)	76.5 (7.8)	72.4 (8.4)	0.50**	76.2 (7.0)	73.2 (9.0)	0.18**	74.9 (7.7)	70.6 (9.9)	0.25**	73.7 (7)	70.7 (8.7)	0.19**	70.8 (7.7)	65 (8.3)	0.73**
No Step Reach (cm)	324.0 (9.6)	317.7 (10)	0.64**	332.8 (8.4)	326.2 (9.7)	0.73**	339.1 (8.7)	334.4 (9.8)	0.50**	345 (6.7)	340 (9)	0.30**	348 (7.1)	341.1 (8.7)	0.39**
Max. Jump (cm)	91.9 (8.7)	86.6 (10)	0.57**	90.8 (8.1)	88 (10.3)	0.15**	88.6 (8.4)	83.6 (10)	0.53**	85.5 (7.5)	82.8 (9.1)	0.17**	81.1 (8.6)	75.8 (8.6)	0.62**
Max. Reach (cm)	339.5 (9.7)	332.1 (11)	0.71**	347.5 (8.6)	341.3 (10.2)	0.66**	350.9 (27)	347.6 (10)	0.16	356.7 (7.3)	352.2 (9.1)	0.27**	358.1 (7.4)	352.5 (8.7)	0.33**
Bench press (rep.)	9.1 (4.5)	9.7 (5.2)	0.14	9.7 (4.8)	10.6 (5.4)	0.18	10.5 (5.0)	11.0 (5.5)	0.10	12.9 (5.2)	12.9 (5.4)	0.01	12.0 (5.0)	12.2 (5.7)	0.04
Lane Agility (s)	11.1 (0.45)	11.4 (0.57)	0.28**	11.2 (0.47)	11.5 (0.68)	0.27**	11.3 (0.49)	11.6 (0.85)	0.20**	11.6 (0.54)	11.9 (0.71)	0.20**	11.9 (0.63)	12.3 (0.68)	0.61**
Sprint (s)	3.2 (0.1)	3.3 (0.16)	0.34**	3.2 (0.13)	3.3 (0.19)	0.20**	3.3 (0.13)	3.4 (0.20)	0.21**	3.3 (0.15)	3.4 (0.18)	0.20**	3.4 (0.17)	3.5 (0.20)	0.54**

*PG, point guard; SG, shooting guard; SF, small forward; PF, power forward; C, center; M, Mean; SD, Standard deviation; rep., repetition; Italics are effect sizes for Man-Whitney *U* test. Legend: ^∗^*p* < 0.05; ^∗∗^*p* < 0.01.*

More specifically, drafted point guard, shooting guard, small forward and power forward were revealed to be heavier than those who were not (*p* < 0.01, Cohen’s *d* for PG = 0.50 and *r* for SG, SF and PF = 0.16–0.19, small effect size). Meanwhile drafted PG, SG, PF and C demonstrated longer standing reaching (*p* < 0.01, Cohen’s *d* = 0.32–0.57, small effect size) and maximum jump reach (*p* < 0.01, Cohen’s *d* for PG and SG = 0.66–0.71 and *r* for PF C = 0.27–0.33, small to moderate effect size) than the latter. Finally, drafted PG (*p* < 0.05, Cohen’s *d* = 0.27, small effect size), SG (*p* < 0.01, *r* = 0.16, small effect size), SF (*p* < 0.05, *r* = 0.19, small effect size) and C (*p* < 0.05, Cohen’s *d* = 0.34, small effect size) were shown to have less body fat than the undrafted counterparts. Supplementary Figures 1, 2 demonstrated the evolution of all analyzed variables for drafted and undrafted players from five playing positions.

Table 3 presents the results of discriminant analysis details for drafted and undrafted players, considering the above statistically different variables within each corresponding playing positions. Wilks’ lambda (Λ) was shown to be statistically significant for the discriminant function of PG (*p* < 0.001, Λ = 0.81, ${\text{η}}_{\text{p}}^{2}$ = 0.07 and $r_{c}^{2}$ = 0.19, moderate effect size), SG (*p* < 0.001, λ = 0.87, ${\text{η}}_{\text{p}}^{2}$ = 0.05 and $r_{c}^{2}$ = 0.13, small to moderate effect size), PF (*p* < 0.001, λ = 0.83, ${\text{η}}_{\text{p}}^{2}$ = 0.06 and $r_{c}^{2}$ = 0.17, small to moderate effect size) and C (*p* < 0.001, λ = 0.81, ${\text{η}}_{\text{p}}^{2}$ = 0.07 and $r_{c}^{2}$ = 0.19, moderate effect size). While the discriminant function did not significantly differentiate the drafted and undrafted SF players (*p* = 0.398, λ = 0.94, ${\text{η}}_{\text{p}}^{2}$ = 0.02 and $r_{c}^{2}$ = 0.06).

**TABLE 3:** Discriminant analysis results and structure coefficients (SC) of combine test measurements for drafted and undrafted players from five playing positions.

**Variables (unit)**	**PG**	**SG**	**SF**	**PF**	**C**
Height (cm)	−0.58^#^	−0.74^#^	0.26	0.38^#^	0.55^#^
Weight (kg)	–0.29	–0.20	–0.04	0.33^#^	*N/A*
Wingspan (cm)	−0.58^#^	−0.69^#^	0.46^#^	0.42^#^	0.44^#^
Standing Reach (cm)	−0.55^#^	−0.68^#^	*N/A*	0.44^#^	0.32^#^
Body Fat (%)	0.54^#^	0.44^#^	−0.67^#^	*N/A*	−0.35^#^
Standing Jump (cm)	–0.10	–0.11	0.28	0.38^#^	0.41^#^
No Step Reach (cm)	−0.53^#^	−0.64^#^	0.53^#^	*F*	*F*
Max. Jump (cm)	−0.30^#^	–0.18	0.48^#^	0.33^#^	0.36^#^
Max. Reach (cm)	*F*	*F*	*N/A*	*F*	*F*
Lane Agility (s)	0.20	0.24	–0.18	−0.46^#^	−0.31^#^
Sprint (s)	0.28	0.13	–0.11	−0.38^#^	0.09
Wilks’ lambda	0.81	0.87	0.94	0.83	0.81
Chi-square (*χ^2^*)	34.49	31.15	9.44	76.89	27.86
Significance	< 0.001	< 0.001	0.398	< 0.001	< 0.001
${\text{η}}_{\text{p}}^{2}$	0.07^∗∗^	0.05^∗^	0.02^∗^	0.06^∗∗^	0.07^∗∗^
$r_{c}^{2}$	0.19^∗∗^	0.13^∗∗^	0.06^∗^	0.17^∗∗^	0.19^∗∗^
Reclassification (%)	68.3	66.4	59.9	68.4	71.4

*^#^Structure coefficient ≥ |0.30|; ^∗^small effect; ^∗∗^moderate effect; *N/A* denotes the variable that was not used in discriminant analysis according to the results of Table 1*missing*; F denotes the variable failing the tolerance test (minimum level = 0.001). Standing Jump, No Step standing vertical Jump; No Step Reach, No Step Vertical Reach; Max. Jump, Maximum Vertical Jump; Max. Reach, Maximum jump reach; Sprint, Three-quarter court sprint.*

The structure coefficients of height and wingspan (SC range: |0.38–0.74|) revealed that they were two common contributors to the discriminant functions of PG, SG, PF and C. Whereas on the other hand, each playing position was emphasized by corresponding position-based variables. The discriminant function for PG had the emphasis on standing reach, body fat%, no step vertical reach and maximum vertical jump (SC range: |0.30–0.55|). The function for SG was emphasized by: standing reach, body fat%, no step vertical reach (SC range: |0.44–0.68|). For PF, the analysis pointed: weight, standing reach, no step standing vertical jump, maximum vertical jump, lane agility and three-quarter court sprint (SC range: |0.33–0.44|). Lastly for C, the model enhanced: wingspan, standing reach, body fat%, no step standing vertical jump, maximum vertical jump and lane agility (SC range: |0.31–0.44|). The distribution of discriminant scores of the function for each playing position is shown in Figure 1. The mean scores of drafted and undrafted players for PG were: −0.40 ± 0.93 and 0.60 ± 1.09; for SG: −0.32 ± 0.92 and 0.47 ± 1.11; for PF: 0.55 ± 0.90 and −0.40 ± 1.08; and for C: 0.55 ± 1.04 and -0.50 ± 1.02. Finally, Supplementary Figure 3 was plotted, using meaningful contributors in each discriminant function to determine the normative profiles for drafted and undrafted players from five playing positions.

## Discussion

The aims of current study were to find differences in combine measurement tests between drafted and undrafted players within large cohort of player samples from NBA, and to describe different anthropometric and physical profiles of different playing positions. The main results demonstrated that drafted players generally outperformed undrafted players in height, wingspan, no step standing vertical jump test, no step vertical reach test, maximum vertical jump test, lane agility test and three-quarter court sprint test. Position-specific variables were further identified that discriminate drafted players from undrafted players. Evaluating players’ fitness is crucial for coaches and team managers so that they could be conscious of the fitness changes and limitations of different positions, optimizing correspondent training practices and player recruitment. To the best of our knowledge, this study is the first to analyze NBA draft combine test performance considering five different playing positions. As NBA represents the highest basketball competitive levels, the findings help to deepen our understanding of talent identification and important prerequisites for being able to play under such circumstance.

The anthropometric and physical characteristics of basketball players are important predictive factors of whether they will reach higher level of this sport as well as demonstrate decent match performance (Köklü et al., 2011; Teramoto et al., 2018). Consistent with previous studies focusing on body size profiles of other basketball leagues or playing levels (Drinkwater et al., 2008; Ferioli et al., 2018; Svilar et al., 2018), our findings reveal that height, weight, wingspan, low body fat are essential attribute prerequisites for being eligible to play in NBA among five playing positions. These fundamental variables allocate players to specific game positions, especially defining the distance between them and the basket (Sampaio et al., 2006). Indeed, taller and heavier players enjoy innate on-court performance advantage over shorter peers (Vaquera et al., 2015), and they are logically standing out from big range of recruiting population during annual draft. Meanwhile, all drafted players were shown to have less percentage of adipose tissue (dead mass) (Garcia-Gil et al., 2018). It means that they possess relatively more fat-free mass (muscle mass) that provide the locomotion and physical performance.

Moreover, partly corroborating the previous studies in that longer arms and upper limb strength were regarded as positive predictors of future on-court performance, especially in shooting and passing related movements (Garcia-Gil et al., 2018; Teramoto et al., 2018), our finding indicated that there was no obvious distinction between drafted and undrafted players in bench press performance. It could be assumed that players participating in the NBA draft have excellent upper limb strength, as well as similar hand characteristics, and hence these variables may not be helpful to determine their draft result.

Furthermore, exclusive of bench press, differences in other fitness tests between drafted and undrafted players of all playing positions have been found. The former jumped higher in vertical jump tests, and finished faster in agility and sprint tests than their counterparts. This indicates that leg power, agility and speed are also reckoned to be fundamental physical attributes qualifying players to enter the league. Playing basketball requires players to develop explosive strength, take-off power, speed and agility so as to make efficient movement during ball possession or out-ball possession (Erculj et al., 2010). Specifically, the execution of high-intensity activities in match such as sprints, jumps, changes of direction, dribbles, screens and hustles are all based on these motor abilities (Drinkwater et al., 2008). In turn, it is shown that the “athletic potential” demonstrated in fitness tests are correlated positively with various match performance indicators like playing time, steals, assists and rebounds (Hoffman et al., 1996; Fort-Vanmeerhaeghe et al., 2016) and distinguish more skilled players from the less skilled ones (Ziv and Lidor, 2009). Indeed, those who possess better-developed physical fitness characteristics that are associated with game-related statistics are favored in terms of further improving match performance in NBA.

Compared with previous study that investigated specific playing roles and athleticism of NBA players (Erčulj and Štrumbelj, 2015), the present study has a main strength of differentiating position-specific key indicators for the combine test result. Although available research has described anthropometric and physical profiles of male basketball players according to different positions (Ben Abdelkrim et al., 2010; Köklü et al., 2011; Ferioli et al., 2018; Svilar et al., 2018), we explored positional determinants leading to successful NBA draft result on the basis of large cohort of players. Results show that anthropometric characteristics (height, wingspan, standing reach, body fat) and leg power (no step vertical reach and maximum vertical jump) served as the key attributes for drafted PG and SG. It could be speculated that for NBA executives, what seems more important when drafting guards is whether these players could have an advantage while jump-shooting, contesting rebounds and blocking shot attempts when they possessed similar competence in quickness and agility (Salador, 2011; Pehar et al., 2017). Previous studies showed offensive and defensive rebounds and blocked shots were key performance indicators that are related to basketball match success (Gómez et al., 2008; Zhang et al., 2018; Dehesa et al., 2019). As it has been widely spreading in NBA where small lineups are competing in the pitch and perimeter players could create more open space and have chances to make three-point shots (Teramoto et al., 2018), guards are consequently required to undertake more defensive works such as contesting for rebounds, deflecting and blocking three-point shots (Man, 2017). Therefore, taller and heavier guards with longer arms and vertical jump height have substantial advantage in those circumstances (Dawes and Spiteri, 2016) and possibly are the drafting targets for teams’ decision-makers.

In contrast, regarding power forward and center, the results revealed that in addition to morphological features and leg power, lane agility and sprint test performance contributed to the differentiation of drafted and undrafted groups as well. It could be explained that first of all, forwards should be as quick and agile as guards because they both performed similar quantity of high activity actions (accelerations, decelerations, jumps and change of directions) during the match (Svilar et al., 2018). At the same time, although traditionally centers are characterized by minor movement frequency and intensity, playing positions have become blurred as multi-dimensional players have emerged during the last years (Man, 2017). Consequently, either forwards and centers are expected to be more versatile their skills, more flexible in playing position. In a word, according to the current development of basketball, they need to amplify their scoring zones from the paint area to three-point line (Zhang et al., 2017). The Wilks’ lambda of discriminant function for SF was not statistically significant, which might suggest that the predictor variables of combine fitness test could not effectively help separate drafted and undrafted players from this playing position.

Although the present study provides valuable information on the key fitness test parameters that contributed to the NBA draft, there are some limitations that need to be acknowledged. Firstly, players’ experience, injury records, match performance in previous leagues and their shooting test results during the daft were not included, which are important factors for decision-makers during player evaluation as well. It is also necessary to note that some players were originally categorized with two playing position, for example, “player *X*” was identified as SG and SF by official draft registration. But the current study only regarded the former as his playing position. Further, given that there are many oversea players that were successfully drafted without participating the combine test, caution should be taken when interpreting the generalizability of the current findings.

## Practical Applications

From the perspective of long-term player development, profiling the NBA draft combine test performance is useful for coaches of youth basketball players while they are transitioning to a more competitive level. Knowing the margins differentiating draft success and failure would help them set practical benchmarks as these players strive to have a professional career. Besides, team executives from lower playing levels are suggested to refine their talent identification based on key fitness determinants of playing positions highlighted in the current findings.

Furthermore, purposeful training programming should be designed for specific positions. Fitness coaches could develop training programs based on the weakness players demonstrated during the combine test, considering specific match-play of each position. Specifically, it is suggested that they focus more attention on the lower-limb strength training of guards via training exercises such as squat, dead lift, contrast strength training and plyometric. While for forwards and centers, exercises highlighting the quick changes of directions should be implemented. This can be integrated into on-court offensive and defensive tactical training, so that their dynamic three-point shooting efficacy and reaction to opponent’s movement would be improved.

## Data Availability Statement

The datasets generated for this study are available on request to the corresponding author.

## Author Contributions

YC, FL, and DB designed the experiments and performed the statistical analysis. YC, SZ, and HL wrote and revised the manuscript. M-ÁG and DB supervised the design and reviewed the manuscript. All authors have made a substantial and direct contribution to manuscript, and approved the final version of the manuscript.

## Conflict of Interest

The authors declare that the research was conducted in the absence of any commercial or financial relationships that could be construed as a potential conflict of interest.

## Footnotes

1 https://stats.nba.com/

2 https://stats.nba.com/draft/combine-anthro/

## Supplementary Material

FIGURE 1 Evolution of NBA Draft Combine Test results for drafted and undrafted players from five playing positions, Part-1.

FIGURE 2 Evolution of NBA Draft Combine Test results for drafted and undrafted players from five playing positions, Part-2.

FIGURE 3 Normative profiles of key determinants of NBA Draft Combine Test drafted and undrafted players from five playing positions.
