# Supplementary figures and images for: Key Anthropometric and Physical Determinants for Different Playing Positions During National Basketball Association Draft Combine Test
Source: Front Psychol. 2019 Oct 22;10:2359. doi: 10.3389/fpsyg.2019.02359 (PMC6820507; doi:10.3389/fpsyg.2019.02359)

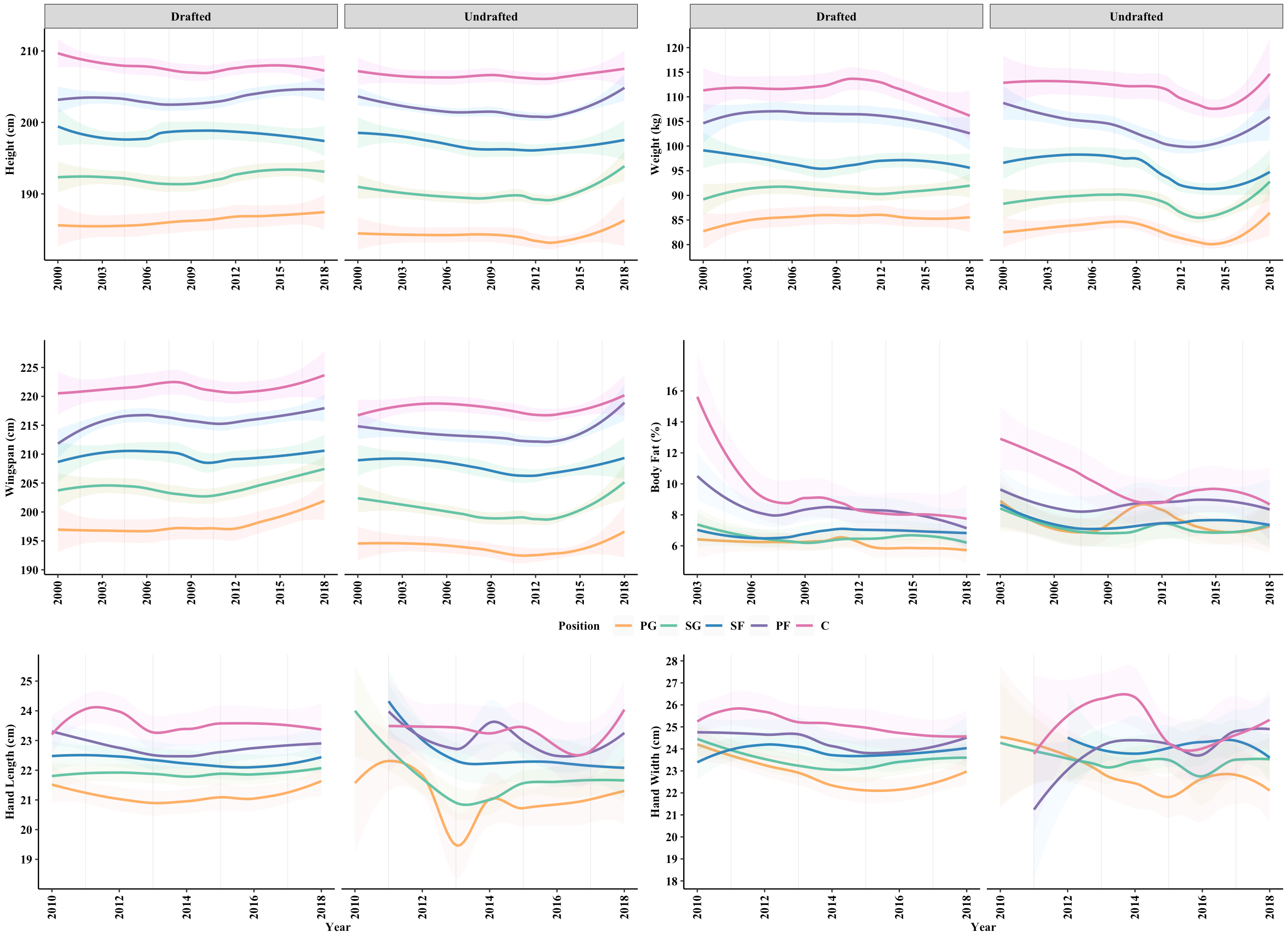

Supplement: FIGURE 1 — Evolution of NBA Draft Combine Test results for drafted and undrafted players from five playing positions, Part-1. [file Image_1.TIFF]

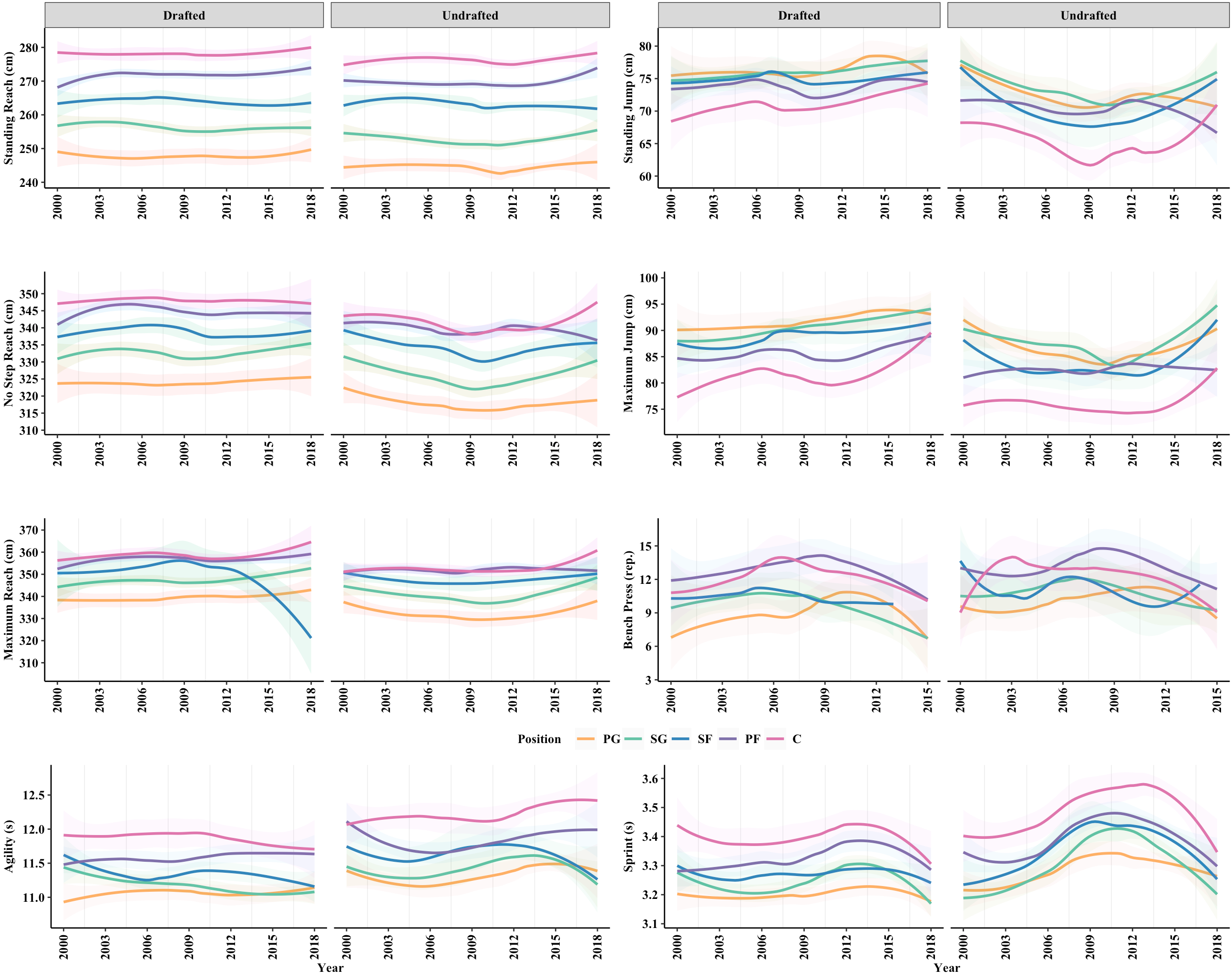

Supplement: FIGURE 2 — Evolution of NBA Draft Combine Test results for drafted and undrafted players from five playing positions, Part-2. [file Image_2.TIFF]

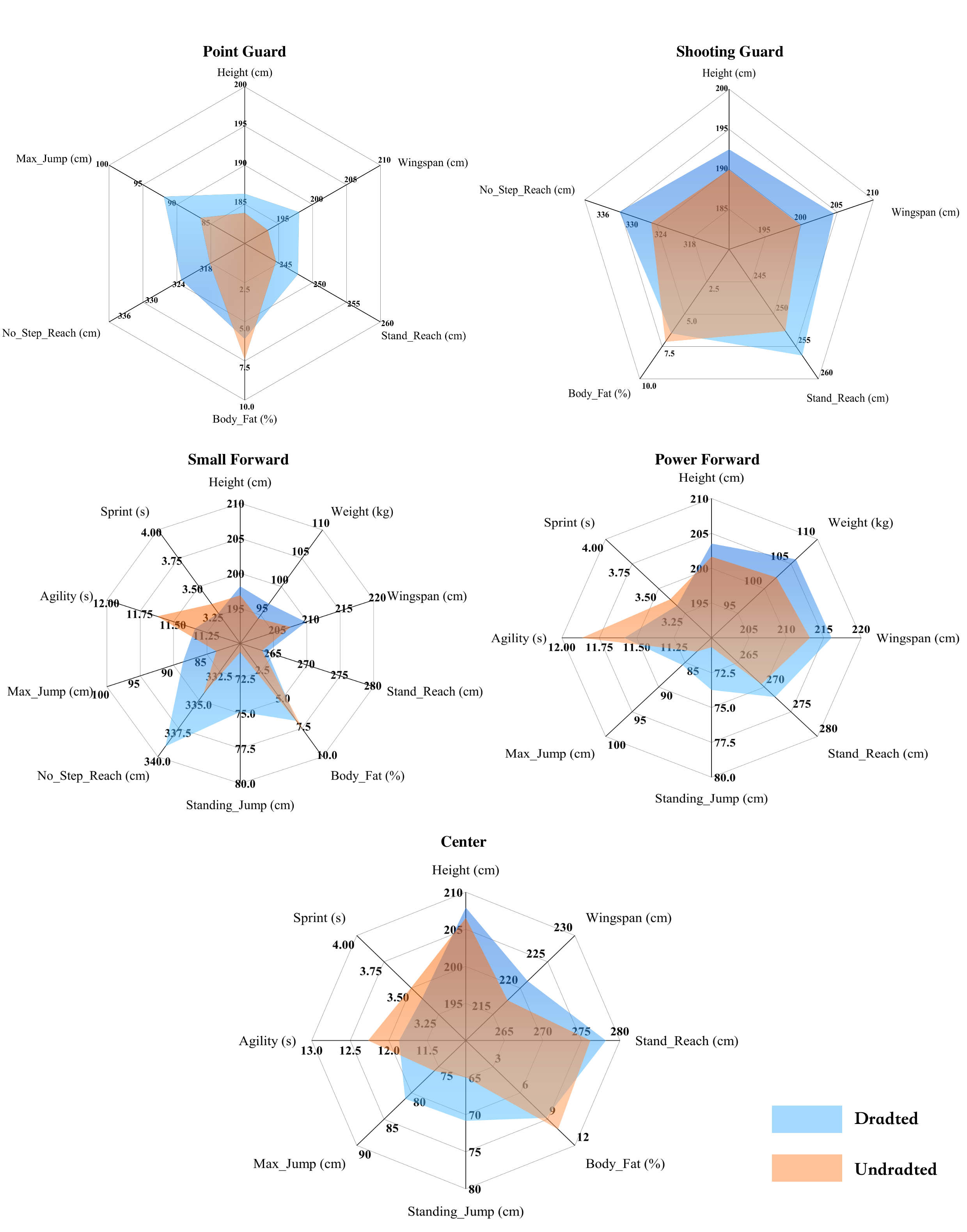

Supplement: FIGURE 3 — Normative profiles of key determinants of NBA Draft Combine Test drafted and undrafted players from five playing positions. [file Image_3.TIFF]
